# ‘I just felt either I’m going to kill someone or I’m going to end up killing myself’. How does it feel to be burnt out as a practicing UK GP?

**DOI:** 10.1080/13814788.2024.2426981

**Published:** 2024-11-25

**Authors:** Ishbel Orla Whitehead, Suzanne Moffatt, Barbara Hanratty

**Affiliations:** a Faculty of Medical Sciences, Population Health Sciences Institute, Newcastle University, Newcastle upon Tyne, UK; b Population and Health Sciences Institute, Newcastle University, Newcastle upon Tyne, UK

**Keywords:** Burnout, primary care, general practice

## Abstract

**Objective:**

To explore how it feels to be a burnt out GP in the NHS.

**Design:**

In depth qualitative interviews with 16 UK GPs with self-declared ‘lived experience’ of burnout.

**Setting:**

United Kingdom Primary Care.

**Results:**

Seven male and nine female GPs described their experiences of burnout to a peer researcher. Themes identified were exhaustion and depersonalisation, mental and physical illness, identity and existential crises, and finally tenacity and resilience. Participants were self-reflective and described distress, shame, stigma, and guilt, including times of suicidal behaviour and isolation due to their burnout.

**Conclusions:**

Burnout threatens a GP’s sense of identity, purpose, and functioning in their lives, and ultimately can be life-threatening. Active listening to GP distress and a system wide approach to managing distress and burnout is urgently required.

## KEY MESSAGES

GP burnout can have life threatening effects on physical, mental, and spiritual health, with long term health and social sequelae.

Despite this, GPs describe resilience, tenacity, and a strong sense of ongoing vocation.

Urgent systemic action is needed to listen to GPs with experience of burnout.

## Introduction

Burnout in UK General Practitioners (GPs) is at crisis levels. A recent survey reported one in five were at highest risk for burnout, and over half were emotionally exhausted [1], figures repeated across the world [2]. Burnout is defined as an occupational phenomenon, resulting in exhaustion, feelings of negativity or cynicism towards the job, and reduced professional efficacy [3]. It leads to retirement and resignation [4], adding to post-pandemic workforce crises and safety concerns [5,6]. Burnout appears to be influenced by, and affect, all aspects of health [7]. The aetiology of burnout is still not fully understood but is likely to be multi-factorial [8,9].

The biomedical model that shapes much of medical practice, has so far been unable to offer a comprehensive and holistic understanding of the burnout phenomenon [10]. While there are studies that quantify burnout [2,11], some exploring underlying factors [12], and many commentaries [13], there is a dearth of qualitative research that allows people with lived experience to share their stories. This means that we have little idea of what it is like for medical doctors, to experience an episode of burnout, rather than mental illness [14]. Understanding this experience for GPs is particularly important given their high burnout rates [15].

This study generated a large dataset from depth interviews with GPs who identify as having experienced burnout, to discuss burnout and wider spiritual health (as defined by GPs [16]) as these concepts appear to be related [1]. In this paper, we ask: how does it feel to be a burned out GP in the NHS?

## Methods

### Study design

This study was grounded within a pragmatic research paradigm, which allows investigation of topics, such as the experience of burnout and spiritual health, and applications of that knowledge.

### Recruitment and selection of participants

Interviewees were recruited from GPs who had completed a survey on burnout and spiritual health [16], and volunteered for an interview. Survey recruitment was via email invitation and social media. Participants needed to have worked in UK general practice, and have personal experience of burnout. Purposive sampling was used to obtain a diverse group of GPs in terms of early, mid, and late career, geography (three of the four UK nations), salaried doctors and partners, and ethnic origin, however, our previous work showed risk of burnout was unrelated to length of service or ethnicity [1]. The survey had 1318 respondents [1], 25 volunteered for interview, 16 took part, and recruitment was stopped at data sufficiency.

### Data collection

A GP researcher (IOW) conducted in-depth video interviews, using Zoom [17], with 16 GPs in the UK between September 2021 and February 2022. An interview guide was developed (Supplement 1), however, interviewees were given space to tell their stories as they wished, in line with the depth interview method. A risk assessment was undertaken (Supplement 2) and participants were encouraged to access support services if needed. Ethics approval was obtained. The topic guide was revised after input from the public. Initially, in discussions before covid, those in our patients and the public group appeared very concerned about GP burnout, and keen for research in this area. However, after the events of 2020, there appeared to be less sympathy with the topic. Voice are a public and patient engagement in research group who were asked to discuss the findings of the preceding survey on GP burnout, and review the interview guide for these interviews. https://voice-global.org/. Interviews lasted from 49 to 75 min. Audio recordings were pseudonymised, then auto-transcribed [18], and corrected by the researcher.

### Analysis

A reflexive thematic analysis was conducted using paper NVivo software, using Braun and Clark’s guide [19,20]. This involved immersion and familiarisation with the data, coding, development of themes, and report writing [19]. Participants were invited to comment on a near-final version of this article.

## Results

Sixteen GPs were interviewed. Table 1 summarises demographic characteristics, and personal burnout scores at the time of the interview. Six participants were experiencing moderate personal burnout and two severe personal burnout. Thirteen participants were white, and one participant’s primary medical qualification was from outside the UK.

**Table 1.** Participant characteristics and total personal burnout score.

Participant	Gender*	Total personal burnout (from Copenhagen burnout inventory) [21]**
1	Man	66.67
2	Man	45.83
3	Man	41.67
4	Woman	91.67
5	Woman	37.50
6	Woman	54.17
7	Woman	16.67
8	Woman	58.33
9	Man	62.50
10	Woman	70.83
11	Woman	37.50
12	Woman	54.17
13	Woman	79.17
14	Man	29.17
15	Man	Not given
16	Man	Not given

* Gender as registered with the UK General Medical Council.

** The Copenhagen burnout inventory consists of nine items across three burnout domains, personal, work and patient. Moderate burnout is suggested by scores of 50–74, high burnout scores 75–99, and 100+ for severe burnout.

**Figure 1.**
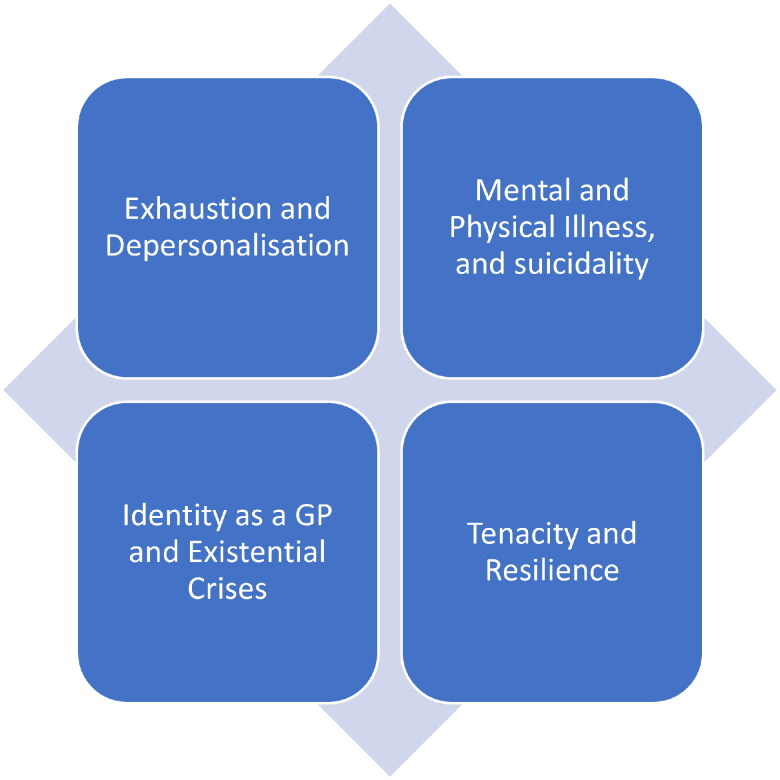
Themes from interviews with GPs.

Throughout the interviews, GPs described multiple facets of their burnout experience. The use of a peer researcher allowed participants to feel understood and empathised with, however, use of a peer will have affected the data given, and added risks to the researcher. The data presented here consider themes related to the question, ‘how does it feel to burnout as a practicing UK GP?’ and are summarised in Figure 1.

### Exhaustion and depersonalisation

Workplace related exhaustion was presented as a primary manifestation of burnout. All participants described fatigue with organisational aspects of work, media coverage of primary care, and exhaustion with their workload. One GP, talking about sick leave, commented that ‘I think for a while I did just sleep’. (GP5). Another likened their experience to chronic fatigue syndrome (GP12).

Participants found depersonalisation a distressing symptom of burnout. They described having so many patients on their lists, they couldn’t see them as humans. The move to telephone and remote consultations during COVID, appeared to worsen depersonalisation.

“You would see literally a list of 80 call-backs and they stopped being people. They just became problems to overcome and solve. And you do start to feel you do lose the empathy.” (GP9)

Depersonalisation appeared to be a secondary phenomenon—a symptom of underlying exhaustion, work stress, difficult team dynamics, co-morbid illness, neurodiversity, or personal stress.

“Almost with aggression towards the patient, you know sort of like “Oh, you know don’t bother me with this” and you know sort of… A lack of empathy I guess. To protect myself. Because I couldn’t bear… because when I did have empathy it would completely go in this other direction” (GP7)

Depersonalisation contrasted with participants’ strong sense of vocation and commitment to patient care. Some participants felt that patients would forgive them the odd ‘bad day’, especially if they’d had a long relationship, but that as things got worse, patient care was affected.

“…there was a period of time … where I became quite bitter, even towards my patients.” (GP14)

### Mental and physical illness

Most participants had taken some time away from work. Some GPs described the moment of crisis meant they could not remain in work, and became emotional recalling this:

“I went into work and my first patient came in and I literally couldn’t hear what she was saying to me it was it was kind of like a curtain had come down and I just. It was terrifying… and the minute I shut the door I started crying and it was almost like I couldn’t stop crying for weeks.” (GP12)

Others described a more insidious onset, that still led to the realisation that things could not continue as they were:

“I said, I’m really sorry, I can’t do it. And that was the day that I stopped work. But in hindsight, I hadn’t been well, probably for several months.” (GP8)

These moments of crisis intertwined with physical and mental illness, and emotional lability, with some experiencing a general decline in all aspects of health over weeks to months. Some participants planned a sabbatical, or retirement, to gain a break, For others, A crisis gave participants ‘permission’ to be off sick, in a way that a slow realisation of burnout did not.

“I was quite determined not to take sick leave. I don’t know, probably at the time I didn’t want the stigma of going off sick.” (GP10)

### Mental illness

Participants were asked how their ‘burnout’ differed from mental illness, especially as many had experienced both. Where physical illnesses and symptoms were felt to indicate impending burnout, mental illness, was described as a consequence of burnout. Those with predisposing or concurrent mental illness articulated that while some features overlap, burnout was related, but different. Burnout appears to affect the core of identity, having greater consequences for the individual, when compared to mental illness:

“It [burnout] was deeper and longer and harder and going to get deeper, more existential.” (GP8)

Burnout was also specifically occupational rather than general, with one participant (GP11) clarifying that they were fine when away from work, and another (GP4) discussed the ‘trudge’ being specifically work related, and while her depression had never required time away from work, burnout made her feel ‘a real sort of “I want to get out I just can’t stand it” that I can’t do anymore’.

Care received from specialist services (such as the Practitioner Health Service) [22] was perceived as helpful, whilst interactions with general mental health services were challenging. The participants who had needed admission to psychiatric wards felt that they were different from other non-medical patients and struggled with the patient role. They perceived stigma and othering for being a mentally unwell doctor. One female participant described a situation when, as an inpatient, she saw a consultant who had been a fellow pupil at a public school. She felt shamed by the implication that this was not a situation for ‘people like them’:

A male participant described visiting his GP while suicidal, and prescribed medication, but not really feeling supported and listened to as a peer. Other concerns related to confidentiality, that GPs could be identified as being ‘different’, which would compromise their anonymity. Participants struggled to identify themselves as patients, sometimes using stigmatising language to distance themselves.

### Physical illness

Participants recognised physical illness, including eight loss, poor sleep, exhaustion, and acute illnesses were all recognised, in retrospect, as being early signs of burnout.

“I got [a physical illness] in the summer before I broke down and I didn’t pay that much attention but actually that was a big red flag really.” (GP8)

Only one participant primarily took time away from work for physical illness. This participant had a lifechanging illness at a relatively young age, yet blamed burnout as the underlying cause of his physical illness.

### Suicidality

Some participants explained they had episodes of feeling suicidal, with others describing attempting to end their lives. Those who had made attempts to end their lives were fully aware of the distress they were under, yet also the distress these attempts caused to their families. While these difficult times were discussed, the emotional lability evident elsewhere in these interviews was absent. Gallows humour was used, and often a matter of fact, clinical tone, by both participant and interviewer, to cover the enormity of the topic. One participant (GP2) described the ease with which he could take actions to end his life, whereas another (GP14) was prevented from attempting to end his life, due to his medical knowledge:

“The inordinate ease [with which] I could have given myself an injection. Because most people sort of think that it’s a difficult thing to do. Well, that that was that was the easy part in some ways, to be quite honest, it was just, draw up ten… ampules into syringe and put a butterfly in. As a [doctor], finding a vein’s not tricky to be quite honest… so in that sense, it wasn’t practically or….emotionally a difficult point.” (GP2)

“And the time I felt very suicidal, I didn’t act on my thoughts because I knew I was 20 minutes away from [hospital]. And the problem with those buggers is, they’d probably save me. I said, there’s no point.” (GP14)

Thoughts of suicide were echoed by other participants who felt that continuing to work while burned out would result in ending their life, although no attempt had been made, yet.

“Literally at that point I just felt either I’m going to kill someone or I’m going to end up killing myself if this goes on. And I just said, I can’t do this anymore.” (GP9)

Attempts to take their own life resulted in interventions from other agencies, such as social services, and the GMC (General Medical Council). GMC involvement was particularly distressing when it occurred, because conditions of regulation are made public on the website, for patients and the public to see. One GP (GP2) described a perception that once the GMC were alerted to mental health issues, a doctor is subject to heightened scrutiny for even minor issues, with little sensitivity to the wellbeing of an individual doctor.

### Identity as a GP and existential crises

Throughout the interviews, participants struggled to reconcile their burnout, and being in the patient role, with their own identity as doctors, especially while away from work. They described feeling stigmatised by their peers treating them, and there appeared to be a taboo of crossing between the roles of patient and doctor.

“Hard to put it into words. You’re definitely treated differently to patients. When I was an inpatient I found it really patronising. A [doctor] said to me “Oh, I have conversations like this with all the patients”, you know, it’s kind of like saying, there’s nothing special here, you know there’s no reason why you should be here, and there’s no reason why you should be depressed…. really unhelpful kind of shaming, almost.” (GP12)

Interviewees had an ideal of a GP in mind, and often a nostalgia for the ‘good old days’. Participants described a disconnect between their expectations of themselves as a GP, and the day-to-day work as a GP. They also expressed some bitterness, looking back at the hardships of training, with the promise of a satisficing career that no longer exists. Burnout challenged participants as to whether they could be the GP they envisioned, whether that was possible in the current system, and whether that was healthy for them. One participant described the mask he wore as a GP before his burnout, and how he now tried to be more human, more authentic, less idealistic, in his presentation.

“When I first qualified as a GP, I went out and bought a jacket with elbow patches. Banana colour chinos and a blue shirt so I look the part and I loved that, I felt really proud to walk into practice: “I look like a GP, I’m a GP!” But now, you know, I take great pride coming into work looking a bit of a mess, sometimes the wearing of the less clinical clothing, it’s a way of sort of saving a bit of myself amongst the job that consumes you.” (GP1)

There was a grief for the life they expected as a GP, a feeling that their reality was not what they anticipated when choosing general practice. One participant (GP14) working in a deprived area described his strong commitment to working in that area. Other interviewees also described a strong sense of vocation towards being a GP and a feeling of being ‘mis-sold’ the promise of the role. The language used was often religious- that of a calling, vocation, a service to others, it being sacrosanct. A participant described growing up witnessing his father’s enjoyment of all aspects of general practice, and the community respect that this afforded. After burnout, he felt his career would be shorter than envisaged. While some of the culture change in general practice was welcomed, there was a layer of regret that participants were unable to fulfil their expected life path as a GP. Interviewees also expressed both guilt and acceptance that after burnout, they would not be able to return to work as before.

“I feel absolutely blessed-to help however many people a day, and people come in and tell you the most secret, confidential things. Feels like a, just a real privileged position, a sacrosanct thing. I never considered not doing it, you know, giving it up. *[tearful]* For the first time this year, I thought, I could do something else, and I don’t need to do this. Why do it?” (GP1)

A sense of failure was implicit, with participants using the term ‘failure’ to describe either their move away from clinical work or the fear of failure being a driver to continue doing some clinical work. Leaving medicine as a career was a source of mixed emotion. People who had planned imminent retirement struggled to articulate what they would do with their time. Future plans were vague, without the hook of identity that being a GP provides. One spoke of an ‘empty canvas’.

Guilt and shame, both implicit and articulated, ran as a thread through all the interviews. Participants felt guilty for being ill as a doctor, for making attempts to end to end their lives, for problems within their home and family life, for providing less than ideal patient care, and for changes to working hours. A sense of failure and guilt contributed to burnout, in an apparent vicious circle.

“For the first time this year, I thought I could do something else, and I don’t need to do this. Why do it? And it pays very well, you know, there’s a guilt associated, [with] that incredibly well-paying job, if you feel like you’re doing a bad job. And you still get paid the same amount, when I feel rubbish and it almost feels morally that I shouldn’t be doing it. And… You know I’ve got rent to pay, children to pay for.” (GP1)

### Tenacity and resilience

Following on from guilt and a strong sense of vocation, keeping GPs in the job, there were examples of tenacity from participants. Describing a career that was embedded in their identity, the idea of leaving the profession caused strong negative emotions as already outlined, but also a response of tenacity and determination. Sometimes this appeared to be rooted in defiant self-preservation. For others, it was built on memories of how much they enjoyed being a GP.

“You’ve got to get through this shit. You’ve got to get through everything. You’ve got to carry on. You’ve got to carry on.. I’m going to the other side of this. I can’t let this be. There’s a line I use on patients, and patients come in to you and they’re depressed and they’ve had that trigger point…I say to myself… “this is not going to be the defining moment of my life,” you know what I mean? The rest of my life is not going to be based on this shit thing.” (GP15)

## Discussion

### Summary of main findings

The experiences of burnout shared by these GPs were distressing. The depth of despair described by doctors who felt exhausted, guilty, ill, stigmatised, in crisis and, for some, suicidal was profound. The analysis presented here considers only the direct experience of how these GPs felt to be burned out. While burnout is experienced as an occupational phenomenon, its effects reach into every facet of well-being, identity, and existence. Participants described how sick leave, and stigma around the patient role interacted with their own sense of failure. Participants told their stories of deep distress with little prompting, and a great deal of reflection on their own emotions and distress during their time of burnout. Despite this level of self-awareness, GPs found it difficult to be in the patient role and seek help early.

Participants described the challenges of being ill as a doctor, and the transgressive nature of going from doctor to patient and back again, feeling stigmatised, guilty, and ashamed. While participants described stigma from others, there also appeared to be some self-stigma regarding their patient role. The involvement of agencies, such as medical regulators or social services compounded these feelings. Specialist services, such as Practitioner Health (a UK service for primary care professionals with mental illness and addictions) were praised by participants.

Exhaustion, emotional lability, depersonalisation, and illness, along with stigma, appeared to create an identity and existential crisis for many participants. All described a strong vocation and tenacity to provide community-based patient care. Their struggles with providing the ideal of general practice, appeared to cause distress, in a way sometimes termed ‘moral injury’ [23]. For some, the chance to reassess their ideals of general practice and reconcile that with their own needs led to a new, invigorated sense of self. For others, that challenge appeared to be an ongoing questioning of who am I?

### Strengths and limitations of this study

A strength of this study is the depth interview method, and peer research, which allowed participants to provide valuable data around sensitive topics, such as suicide, and the guilt and shame around that, albeit limited to those who were capable of being interviewed.

Participants were a heterogeneous group, sampled to give a spectrum of experience rather than to reflect the general GP population, and therefore not representative of all GPs. There is rich data from early, mid and late career GPs, male and female GPs, a variety of socioeconomic backgrounds, ethnicity, and religions. Unfortunately, no participants were recruited who would be considered ‘International Medical Graduates’ (IMGs). This is a limitation of the data, as IMGs are more likely to have regulator input [24], and regulator involvement has been considered a risk for doctors’ suicide [25]. Research on this topic is limited to hearing accounts of GPs that have either not acted on their thoughts to end their lives, or whose lives have not ended with those attempts. However, many GPs do take their own lives [26].

### Comparison with other literature

Some of our findings on the topic of burnout and distress in GP are similar to previous work. It had been noted that there is little good quality data on doctors as patients, and much expert opinion [27]. A narrative review in 2020 found that burnout and stigma are barriers to seeking help, as we also observed [28]. In our data, we noted exhaustion and emotional lability leading to crisis for participants. A qualitative study from 2009 reported that doctors may have a perception of invulnerability to illness, which can be a challenge for doctors as patients, and also for doctors treating doctors [29]. Medical knowledge is proposed as one of the reasons behind completed suicide in doctors [30], as well as a barrier to accessing healthcare [27]. However, whilst much has been written about suicidal GPs [31], GPs have seldom shared their experiences and thoughts of taking their own lives [27]. One of our participants described being kept safe from suicide due to his medical knowledge, and trust in the expertise of his hospital colleagues.

Our data echo the findings from 2017 of a study of 47 in depth interviews with GPs working with mental illness and distress [32]. These interviews elicited similar stories and themes of exhaustion, shame, sense of failure, living with suicidal ideation, and potential normalisation of such unhealthy phenomena in GPs. The study authors highlighted that there should be caution in placing the burden of good holistic health onto the individual GP, and our data would suggest that this is still an issue. An interpretive phenomenological study with general practitioner partners living with distress [14], described the self being subsumed into the doctor role and reclaimed, which was described clearly in our data. Participants in our study were highly motivated to manage their own distress and had tried multiple strategies to seek specialist help. However, the depth of distress, and the shame and stigma of seeking healthcare, prevails. Little appears to have changed in the 5 years since Riley and colleagues described acute mental distress and suicidality in GPs [32]. Our data show that GPs go on being distressed, leave practice, or may attempt to take their own lives.

The experience of burnout involving both a crisis or collapse, and damage to self-image has been described in other studies regarding burnout in other groups [33–35]. The distress of regulatory involvement, and lack of support with that, was also reported by Brooks and colleagues [36]. Our data add an important perspective on the depth of distress, and existential crisis experienced by GPs with burnout.

### Suggested systemic changes

This paper details the very individual experience of an episode of burnout for GPs. However, as burnout is an occupational phenomenon, any intervention must consider the occupational environment. These individuals do not lack resilience and tenacity, in contrast, it is systematic resilience that is required. Many participants noted the benefits of specialist mental health care, provided by the Practitioner Health Service in the UK, however those who had been physically ill, or more severely mentally ill struggled with accessing holistic care. We would suggest that further research into specialist holistic health care for staff, in a biopsychosocialspiritual model is required.

## Conclusion

This study adds to our understanding of GP burnout, with in-depth insights into how GPs feel before and during a burnout crisis, and the holistic impact of a burnout episode on their health. Despite the established association between burnout and resilience in the literature, participants did not lack resilience- many had been through dark experiences and survived to be reflective and articulate advocates of primary care. Our work suggests that enhanced provision of specialised health services (physical, mental, social, and spiritual) and changes in regulatory practices (for example greater support for doctors facing GMC or other services involvement) may tackle the stigma of being a patient and support doctors with burnout and illness. Peer research, such as this can be emotionally taxing, and this should be considered in future research. It seems clear that responsibility for the health of the GP workforce needs to be shifted away from individuals, and consideration given to how GP distress can best be identified and managed at a system level. Further research into the holistic healthcare of staff should be undertaken, including biopsychosocialspiritual provision.

## Ethical approval

Given by Newcastle University on 25th June 2021 Ref: 13413/2020, HRA/REC approval is not required.

## Supplementary Material

Supplemental Material

Supplemental Material

Supplemental Material

## Data Availability

This data will be stored and anonymised data will be available on reasonable request.
