# Supplementary material for: ‘I just felt either I’m going to kill someone or I’m going to end up killing myself’. How does it feel to be burnt out as a practicing UK GP?
Source: Eur J Gen Pract. 2024 Nov 25;30(1):2426981. doi: 10.1080/13814788.2024.2426981 (PMC11590194; doi:10.1080/13814788.2024.2426981)
Supplement: Supplemental Material [file IGEN_A_2426981_SM8325.docx]

**This protocol has regard for the HRA guidance and order of content**

**FULL/LONG TITLE OF THE STUDY**

Keeping up General Practitioners’ spirits during and after the covid pandemic: What are the underlying predisposing, precipitating, perpetuating and protective spiritual health factors in GP burnout using in-depth interviews.

**SHORT STUDY TITLE / ACRONYM**

GP burnout and spiritual health interviews

**PROTOCOL VERSION NUMBER AND DATE**

Version 4 6^th^ July 2021

|  |  |
| --- | --- |
|  |  |
|  |  |

We confirm that the following protocol has been agreed and accepted and that the Chief Investigator agrees to conduct the study in compliance with the approved protocol and will adhere to the principles outlined in the Declaration of Helsinki, the Sponsor’s SOPs, and other regulatory requirement.

I agree to ensure that the confidential information contained in this document will not be used for any other purpose other than the evaluation or conduct of the investigation without the prior written consent of the Sponsor

I also confirm that I will make the findings of the study publically available through publication or other dissemination tools without any unnecessary delay and that an honest accurate and transparent account of the study will be given; and that any discrepancies from the study as planned in this protocol will be explained.

# LIST of CONTENTS

| **GENERAL INFORMATION** | **Page No.** |
| --- | --- |
| HRA PROTOCOL COMPLIANCE DECLARATION | i |
| TITLE PAGE | ii |
| RESEARCH REFERENCE NUMBERS | ii |
| SIGNATURE PAGE | iii |
| LIST OF CONTENTS | iv |
| KEY STUDY CONTACTS | v |
| STUDY SUMMARY | v |
| FUNDING | vi |
| ROLE OF SPONSOR AND FUNDER | vi |
| ROLES & RESPONSIBILITIES OF STUDY STEERING GROUPS AND INDIVIDUALS | vi |
| STUDY FLOW CHART | vii |
| SECTION | |
| 1. BACKGROUND | 1 |
| 2. RATIONALE |  |
| 3. THEORETICAL FRAMEWORK |  |
| 4. RESEARCH QUESTION/AIM(S) |  |
| 5. STUDY DESIGN/METHODS |  |
| 6. STUDY SETTING |  |
| 7. SAMPLE AND RECRUITMENT |  |
| 8. ETHICAL AND REGULATORY COMPLIANCE |  |
| 9. DISSEMINATION POLICY |  |
| 10. REFERENCES |  |
| 11. APPENDICES |  |

# KEY STUDY CONTACTS

| Chief Investigator | Orla Whitehead, Newcastle University, Newcastle upon Tyne NE1 7RU orla.whitehead@newcastle.ac.uk |
| --- | --- |
| Study Co-ordinator | As above |
| Sponsor | Newcastle University Newcastle upon Tyne NE1 7RU |
| Funder(s) | Orla Whitehead is funded via an NIHR In Practice Fellowship |
| Key Protocol Contributors | Professor Barbara Hanratty Population Health Sciences Institute Campus for Ageing and Vitality Newcastle University [barbara.hanratty@newacstle.ac.uk](mailto:barbara.hanratty@newacstle.ac.uk)  Professor Suzanne Moffatt Ridley Building, Newcastle University [suzanne.moffatt@ncl.ac.uk](mailto:suzanne.moffatt@ncl.ac.uk)  Professor Carol Jagger Baddiley Clark Building, Newcastle university carol.jagger@ncl.ac.uk |

**STUDY SUMMARY**

| Study Title | Keeping up General Practitioners’ spirits during and after the covid pandemic: What are the underlying predisposing, precipitating, perpetuating and protective spiritual health factors in GP burnout using in-depth interviews. |
| --- | --- |
| Internal ref. no. (or short title) | Understanding GP burnout and spiritual health via in-depth interviews |
| Study Design | In depth qualitative interviews |
| Study Participants | Volunteer GPs with ‘lived personal experience’ of burnout, and were on the GMC GP register at the time of that experience. |
| Planned Size of Sample (if applicable) | Between ten and twenty one-off interviews. |
| Planned Study Period | July to October 2021 |
| Research Question/Aim(s) | What are the underlying predisposing, precipitating, perpetuating and protective spiritual health factors in GP burnout? How do the phenomena of GP spiritual health and GP burnout interact? Could this interaction lead to lower predisposing, precipitating and perpetuating, or greater protective factors for burnout in GPs? |

**STUDY LAY SYNOPSIS**

In-depth interview study of volunteer UK GPs with self-declared personal experience of burnout, to explore the relationship between burnout and spiritual health already identified through quantitative work, and to answer the following research questions: What are the underlying predisposing, precipitating, perpetuating and protective spiritual health factors in GP burnout? How do the phenomena of GP spiritual health and GP burnout interact? Could this interaction lead to lower predisposing, precipitating and perpetuating, or greater protective factors for burnout in GPs?

**FUNDING AND SUPPORT IN KIND**

| **FUNDER(S)**  (Names and contact details of ALL organisations providing funding and/or support in kind for this study) | **FINANCIAL AND NON FINANCIALSUPPORT GIVEN** |
| --- | --- |
| National Institute for Health Research | In Practice Fellowship for Orla Whitehead |
|  |  |
|  |  |

**ROLE OF STUDY SPONSOR AND FUNDER**

The sponsor is Newcastle University, which will support the study via policies, data storage, software, insurance and indemnity. Newcastle University is the employer of the chief investigator and has overall responsibility for the research. The study supervisors are employed by Newcastle University, and their responsibilities include checking the study is well planned, and good quality, including the literature review of existing evidence already carried out. Newcastle University will provide ethical oversight, and ensure approval before the research starts. Newcastle University will ensure appropriate arrangements are made for making information about the research publicly available before it starts, will agree appropriate arrangements for making data accessible, with adequate consent and privacy safeguards, in a timely manner after it has finished; and ensuring arrangements for information about the findings of the research to be made available, including to participants, in line with the institutional arrangements.

**ROLES AND RESPONSIBILITIES OF STUDY MANAGEMENT COMMITEES/GROUPS & INDIVIDUALS**

**Study Steering Groups**

Patient & Public Involvement Group- This will be held in September 2021, to discuss initial findings, and help formulate the analysis plan.

Qualitative data discussion group- within the ageing research theme, a group of peers to discuss analysis of qualitative data.

Supervisory group- Barbara Hanratty, Carol Jagger, and Suzanne Moffatt are the supervisory group

External advisory group- Carolyn Chew-Graham, Alastair Appleby, Clare Gerada.

**PROTOCOL CONTRIBUTORS**

Supervisory group

| **KEY WORDS:** | Burnout, moral injury, compassion fatigue, spiritual health, spirituality, religion, spiritual wellbeing, saltutogensis, holistic health. |
| --- | --- |

# STUDY TIMELINE

# July 2021- finalising protocol, and obtaining required ethical and other approvals, insurance etc. Finalise interview topic guide, and pilot interviews.

# August 2021- recruitment, contact participants., and scheduling interviews

September and October 2021- conduct interviews- max 3 interviews per week, discuss initial data at PPI group, and qualitative data group.

November and December 2021- analysis and writing up of paper

January 2021 onwards- submit to journals, and abstract to conferences.

**STUDY PROTOCOL**

Keeping the spirits up of General Practitioners during and after the covid pandemic: What are the underlying predisposing, precipitating, perpetuating and protective spiritual health factors in GP burnout using in-depth interviews.

# 1 BACKGROUND

Burnout is a term used to describe a form of psychological distress associated with work. While burnout leads to physical and mental health sequalae, burnout is not itself considered to be mental or physical illness.^1^ The World Health Organisation describes burnout not as an illness, but as an occupational phenomenon, resulting in exhaustion, mental distance or feelings of negativity or cynicism towards the job, and reduced professional efficacy.^1^ Others describe a loss of meaning in work, and objectification of patients and their families, rather than engaging with their humanity.^2^ Definitions of burnout and moral injury mirror those given by GPs themselves of the term ‘spiritual health’.^3^ Christina Maslach argued that burnout is “erosion of the soul.”^4^ Burnout syndrome is often described as overlapping the concept of “moral injury”, a term taken from military personnel returning from battle, where realties conflict with our moral and ethical code.^5^ Living out an ethical code was also included in the meaning of the term ‘spiritual health’ by GPs.^3^ Holistic health includes spiritual health, and burnout arguably fits better within a ‘spiritual health’ paradigm than mental health. From initial analysis of a survey of GPs, there appears to be correlation between all domains of burnout from the Maslach Burnout Inventory, and spiritual domains from the FACIT-NI spiritual wellbeing score.

The Covid-19 crisis has brought concerns about burnout in doctors to the fore.^6^ Burnout was already thought to be contributory to the current workforce crisis in the UK, as well as higher rates of hazardous drinking and suicide among doctors.^7^ General Practitioners (GPs) are particularly vulnerable to burnout^8^, partly due to the current recruitment and retention difficulties.^7 8^ Covid-19 has amplified difficulties, and a recent survey by the researcher yielded 1300 respondents within eight weeks showing GPs have a high level of concern. It is extremely timely, and important, to explore the relationship between burnout and spiritual wellbeing during the pandemic, to inform and protect the workforce during and after this crisis.

General Practitioners (GPs) are particularly vulnerable to burnout.^8^ GPs are proud to be holistic practitioners, treating the whole person and their family from cradle to grave. However, anecdotally, there appears to be a stigma towards spiritual health needs. Literature discussing how doctors should address their patients’ spiritual health often mention that self-awareness of spiritual health, needs, and distress, are key to be able to begin to meet patients’ needs in this area^10-14^. In a recent survey of GPs in England, 69% described themselves as ‘a spiritual person’, with 48% also describing themselves as ‘a religious person’. These GPs defined spiritual health as self-actualisation and meaning, transcendence and relationships beyond the self, and expressions of spirituality. Self-actualisation included concepts of being able to follow their personal ethical and moral codes. Meaning included having personal meaning to life and relationships. Transcendence included concepts of a spiritual sphere, a concept of a soul, and relationships with communities, friends, family, nature and/or the divine. Expressions of spirituality included both personal and group religious and spiritual practice, for example attendance at church, religious meetings, meditation, prayer and mindfulness practice. Burnout definitions contrast GP definitions of spiritual health, as mental distance from others and the job, negativity and lack of meaning in the job, and lack of relationships with patients and their families. Spiritual health has been linked with reduced risk of burnout in other groups.^15-17^ The connection identified in the aforementioned quantitative survey between spiritual wellbeing and burnout needs further in-depth exploration using qualitative research, to allow better understanding of this phenomenon to develop interventions that could mitigate the high risk of GP burnout throughout the next phase of the Covid-19 pandemic and afterwards.

# 2 RATIONALE

A recent quantitative survey undertaken by the applicant has indicated that GP burnout and spiritual health could be associated. Further qualitative work, via in-depth interviews, will allow this concept to be explored, in order to understand whether there are aspects of spiritual health that contribute to predisposing, precipitating, perpetuating and protective factors for GP burnout. This information is anticipated to inform interventions to prevent and manage burnout in GPs, and potentially assist with rehabilitation back into the workforce after burnout.

**3 THEORETICAL FRAMEWORK**

This a pragmatic piece of work that seeks to explore the experience of burnout and spiritual health with individual GPs with experience of burnout. Neither burnout nor spiritual health will be formally defined for participants, and these terms will be left open for their own experiences and definitions.

# 4 RESEARCH QUESTION/AIM(S)

What are the underlying predisposing, precipitating, perpetuating and protective spiritual health factors in GP burnout using depth interviews.

**4.1** **Objectives**

- To identify the predisposing, precipitating, perpetuating and protective spiritual health factors in GP burnout
- To explore individual and organisational effects on these spiritual health factors, and their effect on burnout

**4.2 Outcome**

To provide data to inform the development of interventions and organisational strategies to prevent, and mitigate burnout in GPs, and aid rehabilitation.

# 5 STUDY DESIGN and METHODS of DATA COLLECTION AND DATA ANALYIS

**Initial screening questionnaire-** Participants will be asked their demographic details, the Maslach burnout inventory, and the FACIT-Sp-NI prior to the interview.

**In-depth Interviews**- The interview guide is attached, with open questions around the three domains of burnout, three domains of spiritual health, with a thread of looking for predisposing, precipitating, perpetuating and protective spiritual health factors throughout the interview. Data will be analysed using interpretative phenomenological analysis methodology, and reflexive thematic analysis, with the researcher keeping a reflective diary throughout the process. Voice to text, and Nvivo software will be used for managing the data, however coloured pens, scissors and paper will also be used for analysis. It will be stored securely on Newcastle University servers. Data will be archived in line with Newcastle University policies.

# 6 STUDY SETTING

- Participants have contacted the researcher after participating in a quantitative survey, or seeing a social media advert, and indicated their willingness to take part in an in-depth interview on the topic.
- Participants will be offered telephone, video call, or if practical, face to face or walking interview.

**7 SAMPLE AND RECRUITMENT**

**7.1 Eligibility Criteria**

Participants will have identified themselves as having ‘lived personal experience’ of burnout, while registered on the GMC GP register.

**7.1.1 Inclusion criteria**

- Current or past registration on the GMC GP register (so post CCT fully registered GPs)
- Would describe themselves as having ‘lived personal experience’ of burnout.
- Were working in the UK as a GP when the participant experienced burnout. This includes roles outside the practice ‘norm’ for example, a GP working in a sexual health clinic, hospice, A&E or OOH would be included if they felt they were working as an autonomous GP.
- Have taken part in a quantative survey on burnout and indicated their willingness to take part in an in-depth interview

**7.1.2 Exclusion criteria**

- Those who were not working in a GP role at the time of their experience of burnout, for example they were a trainee, working as a junior doctor equivalent, or as a staff grade/speciality doctor.
- Were working outside of the UK at the time of their experience of burnout
- No exclusion based on sex, age, ethnicity, disability, maternity, etc.

**7.2 Sampling**

Email invites to a quantitative survey on burnout and spiritual health were sent out widely throughout the UK, to clinical commissioning groups, practices, health boards, clinical research networks and shared via professional networks, and social media. Participants with experience of burnout then were invited to email to be informed of further opportunities, and further email invites were sent.

**7.2.1 Size of sample**

It is expected that the sample will be about 15 interviews, however interviews will take place until data saturation.

**7.2.2 Sampling technique**

Purposive sampling will be undertaken, aiming to gain a range of views from GPs who have volunteered by taking a diverse sample of age, working life, ethnicity, and religious background.

**7.3 Recruitment**

Participants recruited have self identified as having personal experience of burnout after a survey, or after seeing a social media advert, and contacted the PI to express interest in further work.

**7.3.1 Sample identification**

Participants have self identified as eligible. Eligibility will be confirmed at a screening email, where initial consent will be sought. No payment for time will be offered, and distance options for interview will be given.

**7.3.2 Consent**

Consent will be an ongoing process- participants have implicitly consented to contact by contacting the researcher themselves, and disclosing their status as GPs with experience of burnout. They will be invited to participate in an in-depth interview, those who express an interest will be contacted with details of consent, the nature and objectives of the study, and possible risks. They will be given the written consent information assuring anonymity. Capacity to consent will be assumed, but if the researcher has any reason to doubt capacity at the time of the interview, the interview will not go ahead. Participants will be encouraged to ask questions before the interview starts, and to stop and ask at any time during the interview.

# 8 ETHICAL AND REGULATORY CONSIDERATIONS

Ethical approval was sought and obtained from Newcastle University on 25/06/021

Clarification will be sought from the HRA whether further HRA approval is required for recruitment to the study.

## **8.1 Assessment and management of risk**

Potential risks of the study:

| Potential risk | Risk management |
| --- | --- |
| Risks to the researcher if undertaking interviews alone in person, including covid risks | The researcher is experienced in risk assessing home visits, and managing according to lone worker guidance. The location of the researcher, and expected start and finish times will be shared before any visits. The researcher has de-escalation trained, and managing violence and aggression training. If the researcher has any concerns for her safety or that of participants, the interview will be abandoned, and help sought via emergency services if needed. The supervisory team are there to support decision making, and to allow supervision of the health of the researcher. The researcher is trained in use of supervision for issues of transference and counter transference and similar issues that occur when discussing difficult topics. The researcher will wear a face covering and ensure hand hygiene and distancing for covid risk. The researcher is full vaccinated. |
| Risks to the participant | Burnout and spiritual health can be emotive topics to discuss. Consent will be an ongoing process, and should the participant need a break, or to stop at any time, this will happen. The participant will be supported to access signposted resources and support services if need be. If the researcher has concerns about safeguarding the participant if they are vulnerable, or about managing the risk, the supervisory team are there to support decision making. |
| Risks to others | While discussing burnout, and other health issues in GPs, it may become apparent that there is a risk to others (for example patients). If the researcher is concerned about risks to others, these will be discussed with the supervisory team, and decisions regarding this will be shared, with the care of the patient being our first concern. If there are concerns, the participant may be encouraged to take action themselves as a first line, before any undertaking of information sharing, however if there is immediate risk to others, confidentiality may need to be broken in order to safeguard others. The researcher is familiar with these sorts of safeguarding dilemmas, and is level 3 trained. These decisions will not be taken alone, unless an emergency, and will be shared with the supervisory team, and if appropriate, the researchers medical defence organisation. |

**8.2 Research Ethics Committee (REC) and other Regulatory review & reports**

The participants are UK GPs, whether employed or not, and so are recruited by basis of their qualification, and not working status. Newcastle University ethical approval was sought and gained on the 25^th^ June 2021, and NHS REC approval is not required.

**Regulatory Review & Compliance**

- Before Newcastle University can enrol patients into the study, the Chief Investigator will ensure that all appropriate approvals are in place. Newcastle University is the only study site.
- For any amendment to the study, the Chief Investigator, in agreement with the sponsor will submit information to the appropriate body in order for them to issue approval for the amendment.

Amendments

All amendments will be handled in line with Newcastle University policies.

**8.3 Peer review**

Orla Whitehead (Chief investigator) applied for competitive funding for her post via the NIHR. This research project was presented, and received peer reviewed, and was deemed fundable.

**8.4 Patient & Public Involvement (PPI)**

The primary stakeholders in this research is GPs themselves, and the research is GP led with GP participants. The importance of the study topic and questions were raised by discussion at the PPI (VOICE north) group on a previous research project about spiritual health. The group were very concerned about workforce shortages and pressures, and the risks of GP burnout. They also recognised the need to ‘keep spirits up’. This prompted the chief investigator to develop the research project proposed. A PPI meeting with VOICE north is planned for September 2021, to discuss the findings from the quantitative work before this, and the initial qualitative data from the first few interviews. Based on this meeting, it is planned that the interview guide can be developed further, and the PPI group can inform the analysis of the interview data.

**8.5 Protocol compliance**

- Accidental protocol deviations can happen at any time. They must be adequately documented on the relevant forms and reported to the Chief Investigator and Sponsor immediately.
- Deviations from the protocol which are found to frequently recur are not acceptable, will require immediate action and could potentially be classified as a serious breach.

###

**8.6 Data protection and patient confidentiality**

Patient confidentiality will be maintained, and the study will be compliant with all GDPR regulations according to Newcastle University guidance. Data will only be collected that is proportionate, and necessary for the research. Identifiable information will be kept separately from interview data.

8.7 Indemnity

Newcastle University (the sponsor) will provide indemnity for the study, in line with its usual procedures.

**8.8 Access to the final study dataset**

The study team (the CI and supervisory team) will be the only individuals with access to the full data set, and other investigators to check the integrity of the research only if a formal request is received, justified and accepted by the study team. It is not envisaged that secondary analysis will be approved.

### 9 DISSEMINIATION POLICY

### 9.1 Dissemination policy

- - The data will be owned by Newcastle University, who employ the chief investigator.
  - On completion of the study, the data will be analysed and tabulated and a Final Study Report prepared.
  - The full study report will be available from Orla Whitehead
  - Orla Whitehead and the supervisory team will publish the data
  - The NIHR (as funder of Orla Whitehead’s post) will be acknowledged with the standard wording.
  - The study abstract will be shared with participants, and a copy of the final published report
  - The study protocol, full study report, anonymised participant level dataset, and statistical code for generating the results will not be made publicly available.

**9.2 Authorship eligibility guidelines and any intended use of professional writers**

Orla Whitehead intends to be the first and corresponding author, with Suzanne Moffatt, Carol Jagger and Barbara Hanratty as authors. Contributorship statements will be completed.

### 10 REFERENCES

# References (of literature cited in preceding sections)

1. World Heath Organisation. Burn-out an "occupational phenomenon": International Classification of Diseases 2019 [Available from: <https://www.who.int/mental_health/evidence/burn-out/en/>.

2. Fred HL, Scheid MS. Physician Burnout: Causes, Consequences, and (?) Cures. Texas Heart Institute journal 2018;45(4):198-202. Doi: 10.14503/THIJ-18-6842

3. Whitehead IO. Discussing spiritual health in primary care in England. Innovation in Aging 2019;3(Supplement_1):S884-S84. Doi: 10.1093/geroni/igz038.3237

4. Maslach C, Leiter MP. The Truth About Burnout: How Organizations Cause Personal Stress and What to Do About It: Wiley 2008.

5. Talbot SG, Dean W. Physicians aren’t ‘burning out.’They’re suffering from moral injury. Stat 2018

6. Rimmer A. Covid-19: Two fifths of doctors say pandemic has worsened their mental health. BMJ 2020;371:m4148. Doi: 10.1136/bmj.m4148

7. Jones D, Davies P. Burnout in general practice. Innovait 2016;9(6):364-69.

8. Hall LH, Johnson J, Watt I, et al. Association of GP wellbeing and burnout with patient safety in UK primary care: a cross-sectional survey. British Journal of General Practice 2019;69(684):e507. Doi: 10.3399/bjgp19x702713

9. Launer J. Burnout in the age of COVID-19. Postgraduate Medical Journal 2020;96(1136):367. Doi: 10.1136/postgradmedj-2020-137980

10. Vermandere M, De Lepeleire J, Smeets L, et al. Spirituality in general practice: a qualitative evidence synthesis. British Journal of General Practice 2011;61(592):e749.

11. Puchalski CM. The FICA Spiritual History Tool #274. Journal of Palliative Medicine;17(1):105-6.

12. Kligler B, Koithan M, Maizes V, et al. Competency-based evaluation tools for integrative medicine training in family medicine residency: a pilot study. BMC Medical Education;7:7.

13. Anandarajah G, Hight E. Spirituality and medical practice: using the HOPE questions as a practical tool for spiritual assessment. American Family Physician;63(1):81-9.

14. Isaac KS, Hay JL, Lubetkin EI. Incorporating Spirituality in Primary Care. J Relig Health 2016;55(3):1065-77. Doi: 10.1007/s10943-016-0190-2

15. Hardiman P, Simmonds JG. Spiritual well-being, burnout and trauma in counsellors and psychotherapists. Mental Health, Religion & Culture 2013;16(10):1044-55. Doi: 10.1080/13674676.2012.732560

16. Kim HS, Yeom HA. The association between spiritual well-being and burnout in intensive care unit nurses: A descriptive study. Intensive Crit Care Nurs 2018;46:92-97. Doi: 10.1016/j.iccn.2017.11.005 [published Online First: 2018/04/08]

17. Doolittle BR, Windish DM, Seelig CB. Burnout, coping, and spirituality among internal medicine resident physicians. Journal of graduate medical education 2013;5(2):257-61.
