# Supplementary material for: ‘I just felt either I’m going to kill someone or I’m going to end up killing myself’. How does it feel to be burnt out as a practicing UK GP?
Source: Eur J Gen Pract. 2024 Nov 25;30(1):2426981. doi: 10.1080/13814788.2024.2426981 (PMC11590194; doi:10.1080/13814788.2024.2426981)
Supplement: Supplemental Material [file IGEN_A_2426981_SM8324.docx]

## **8.1 Assessment and management of risk**

Potential risks of the study:

| Potential risk | Risk management |
| --- | --- |
| Risks to the researcher if undertaking interviews alone in person, including covid risks | The researcher is experienced in risk assessing home visits, and managing according to lone worker guidance. The location of the researcher, and expected start and finish times will be shared before any visits. The researcher has de-escalation trained, and managing violence and aggression training. If the researcher has any concerns for her safety or that of participants, the interview will be abandoned, and help sought via emergency services if needed. The supervisory team are there to support decision making, and to allow supervision of the health of the researcher. The researcher is trained in use of supervision for issues of transference and counter transference and similar issues that occur when discussing difficult topics. The researcher will wear a face covering and ensure hand hygiene and distancing for covid risk. The researcher is full vaccinated. |
| Risks to the participant | Burnout and spiritual health can be emotive topics to discuss. Consent will be an ongoing process, and should the participant need a break, or to stop at any time, this will happen. The participant will be supported to access signposted resources and support services if need be. If the researcher has concerns about safeguarding the participant if they are vulnerable, or about managing the risk, the supervisory team are there to support decision making.  The text of the written support signposting is given below:  “Thank you very much for your time, we are grateful for your responses.  If you need further support, here are some suggested resources:  NHS Practitioner Health is a confidential service for NHS doctors. https://www.practitionerhealth.nhs.uk/  The Doctors' Support Network is a peer support service. https://www.dsn.org.uk/  A list of support services for GPs can be found here: https://www.rcgp.org.uk/training-exams/practice/gp-wellbeing/wellbeing-help.aspx  Thank you.” |
| Risks to others | While discussing burnout, and other health issues in GPs, it may become apparent that there is a risk to others (for example patients). If the researcher is concerned about risks to others, these will be discussed with the supervisory team, and decisions regarding this will be shared, with the care of the patient being our first concern. If there are concerns, the participant may be encouraged to take action themselves as a first line, before any undertaking of information sharing, however if there is immediate risk to others, confidentiality may need to be broken in order to safeguard others. The researcher is familiar with these sorts of safeguarding dilemmas, and is level 3 trained. These decisions will not be taken alone, unless an emergency, and will be shared with the supervisory team, and if appropriate, the researchers medical defence organisation. |
