# Supplementary material for: ‘I just felt either I’m going to kill someone or I’m going to end up killing myself’. How does it feel to be burnt out as a practicing UK GP?
Source: Eur J Gen Pract. 2024 Nov 25;30(1):2426981. doi: 10.1080/13814788.2024.2426981 (PMC11590194; doi:10.1080/13814788.2024.2426981)
Supplement: Supplemental Material [file IGEN_A_2426981_SM8323.docx]

Burnout and spiritual health interview Guide

One hour

Purpose and introduction-

Checking consent, time, space, withdrawal if needed. Introduction of self, participant, and topic.

Questions-

Open questions:

What is or was your experience of burnout?

Depersonalization

Emotional exhaustion

Personal accomplishment

How do you feel your spiritual health was during that time?

Explore precipitating, predisposing, protective and perpetuating factors

Individual

Organisational

Meaning and purpose, soul, peace

Communities, transcendence, relationships

Religious/spiritual practice, faith

Sources of hope, meaning, comfort, strength, peace, love and connection

How do you think those who **don’t** burnout do it?

Self-description/identity, asking about ethnicity, religion, culture etc.

Closed questions:

Gender as GMC

Ethnicity as census

Religion as census.

Working status (partner, salaried, locum and sessions) before and after burnout episode

Country of primary medical education, GP training

Number of yrs qualified as GP

Thanks, and offer of ongoing support- signposting to practitioner health, GP, BMA counselling etc.
